# Long-term survival among colorectal cancer patients in Finland, 1991–2015: a nationwide population-based registry study

**DOI:** 10.1186/s12885-022-09460-0

**Published:** 2022-04-02

**Authors:** Tobias Olenius, Laura Koskenvuo, Selja Koskensalo, Anna Lepistö, Camilla Böckelman

**Affiliations:** Department of Gastroenterological Surgery, University of Helsinki and Helsinki University Hospital, Meilahden Tornisairaala, PO Box 340, 00029 HUS, Helsinki, Finland

**Keywords:** Colorectal cancer, Survival, Prognosis, National registry study, Finland

## Abstract

**Background:**

Colorectal cancer (CRC) incidence in Finland has risen steadily. Given development in cancer treatments in recent decades, disease-specific data on the long-term prognosis of patients may be obsolete. Thus, this study aimed to report 5-year disease-specific survival (DSS) and relative survival based on tumour spread and site among CRC patients diagnosed between 1991 and 2015 in Finland.

**Material and methods:**

We conducted a population-based registry study among 59 465 CRC patients identified from the Finnish Cancer Registry.

**Results:**

The 5-year DSS for all CRC patients was 56.7% [95% confidence interval (CI) 56.3–57.1%] for 1991 through 2015. Tumour site-specific survival has improved for the period 2006–2015 versus 1991–2005 for right-sided colon cancer from 54.8% (95% CI 53.8–55.8%) to 59.9% (95% CI 58.7–61.1%), for left-sided colon cancer from 54.1% (95% CI 52.9–55.3%) to 61.0% (95% CI 59.8–62.2%) and for rectal cancer from 53.6% (95% CI 52.2–55.0%) to 62.3% (95% CI 61.3–63.3%). The 5-year relative survival for the period 2006 through 2015 was 93.6% for localised disease (stage I); 84.2% for locally advanced tumour invading adjacent structures (stage II); 68.2% for regional disease with regional lymph node metastases (stage III); and 14.0% for metastatic disease (stage IV).

**Conclusions:**

This study confirms that survival for CRC has improved in recent decades in Finland, mirroring observations from other Western countries. However, the classification of tumour spread within the Finnish Cancer Registry differs slightly from the TNM classification, thereby limiting the generalisability of these results.

**Supplementary Information:**

The online version contains supplementary material available at 10.1186/s12885-022-09460-0.

## Background

After prostate and breast cancers, colorectal cancer (CRC) is the third most common cancer in Finland with more than 3500 new cases occurring in 2018 [1]. The 2018 report from the Finnish Cancer Registry (FCR) indicated that the incidence of colon cancer has steadily increased, with age-standardised incidence rates of 42.3/100 000 for men and 34.5/100 000 for women. In comparison, the incidence rate for rectal cancer has remained quite steady with a rate of 29.8/100 000 for men and 16.7/100 000 for women [2].

FCR is a population-based registry cataloguing data for each cancer case in Finland since 1952. The completeness of the data for CRC is 97.4%, exceeding 99% for all solid tumours [3, 4]. The cancer classification used by FCR differs from the TNM classification outlined by the Union for International Cancer Control (UICC), rendering possible the registration of cancer cases lacking complete information on TNM staging [5]. Furthermore, every Finnish citizen has a unique social security number, allowing researchers to combine data on death, follow-up, hospital care periods and treatment using databases from FCR and the Finnish Institute for Health and Welfare.

Previous studies on stage-specific survival for CRC in Finland either relied on small and local study populations or are dated. In a large cohort study in southwest Finland from 1971 to 1990 with a population of 433 000, an increasing age-adjusted incidence for colon cancer reaching 13.5/100 000 for men and 13.1/100 000 for women by 1990 was noted, compared to 11.1/100 000 for men and 7.1/100 000 for women for rectal cancer [6]. No remarkable changes were noted in the spread (Dukes A–D classification) of CRC at diagnosis during this 20-year time period. A slight reduction in rectal cancer mortality was observed, although mortality for colon cancer increased [6]. However, in a more recent study, the relative 5-year survival for colon cancer improved over time from around 20–30% to 50–60% depending upon the region during the period from 1953 through 2002 [7]. In addition, the difference in relative survival between regions and age groups has also narrowed in Finland [7]. Storm et al. studied survival trends among cancer patients in the Nordic countries from 1964 to 2003, summarising Nordic cancer studies and results from the NORDCAN database (a database with comparable information from Nordic cancer registries). They concluded that there is room for improvement in survival for CRC in all Nordic countries [8]. In Sweden, for example, where national guidelines were introduced 2008, survival has improved in recent years [9].

In this nationwide registry study, we aimed to report 5-year disease-specific survival (DSS) and relative survival according to tumour spread and tumour site among CRC patients diagnosed between 1991 and 2015.

## Methods

### Patients

Our study comprises all patients diagnosed with CRC in Finland from 1991 through 2015. We requested and received information about patients (gender and age at diagnosis), tumour-specific data (spread and localisation) and time and cause of death data from FCR. Causes of death were coded using the International Classification of Diseases, tenth edition (ICD-10), and considered disease-specific when recorded as C18–C20 (malignant neoplasm of colon, rectosigmoid junction or rectum). We divided data into two main periods according to the time of diagnosis – the former, 1991 through 2005 and the latter, 2006 through 2015 – as well as into five smaller time periods: 1991–1995, 1996–2000, 2001–2005, 2006–2010 and 2011–2015.

Out of 59 896 cancer cases in total, we identified 59 465 individual patients (that is, 431 patients had cancer two or more times; Supplementary Fig. 1). Among patients experiencing a secondary or tertiary cancer, we only included the initial cancer diagnosis. We also excluded patients with appendix cancer (C18.1; *n* = 1180) and patients with missing information on the diagnosis (*n* = 30). One patient was excluded because of a missing date of death.

### Variables

FCR registers CRC using their own classification, which is not entirely comparable to the traditional TNM staging classification (Table 1) [10]. We received information on the tumour stage based on FCR’s classification as follows: 0) unknown; 1) localised; 2) nonlocalised with only regional lymph node metastases; 3) metastasised further than to regional lymph nodes or invasion to adjacent tissues; 4) nonlocalised with no information on extent; 5) locally advanced with the tumour invading adjacent tissues; and 6) nonlocalised with distant lymph node metastases as well.

**Table 1:** The Finnish Cancer Registry classification system and its relation to UICC TNM^a^ staging (8th edition)

**FCR classification**		**UICC TNM**			
	**Definition**	**T**	**N**	**M**	**Stage**
0) Unknown	The extent of cancer cannot be assessed	TX	NX	MX	
1) Localised	Tumour in situ or tumour invades submucosa or muscularis propria	Tis, T1, T2	N0	M0	0–I
2) Nonlocalised with only regional lymph node metastases	Metastasis to the local lymph node(s). Primary tumour can be localised or locally advanced, but no distant metastases are found	Any T	N1–2	M0	III
3) Metastasised further than to regional lymph nodes or invasion to adjacent tissues	More detailed information on tumour spread has not been reported to FCR. This class was used when marked on an old paper clinical form and no more specific information was available				II–IV
4) Nonlocalised with no information on extent	Metastasis to the local lymph node(s), but distant metastasis cannot be assessed	Any T	N1–2	MX	III–IV
5) Locally advanced with the tumour invading adjacent tissues	Tumour invades through the muscularis propria into the subserosa or pericolorectal tissues; penetrates to the surface of the visceral peritoneum; or directly invades or is adherent to other tissues. No local lymph node metastasis or distant metastasis detected	T3–4	N0	M0	II
6) Nonlocalised with distant lymph node metastases as well	Distant metastasis	Any T	Any N	M1	IV

*Abbreviation*: ^a^Union for International Cancer Control Tumour (T), Node (N) and Metastasis (M)

Tumour location was analysed separately for the right colon, left colon and rectum. The right colon constitutes the caecum to the transverse colon and the left colon starts at the splenic flexure of the colon [11]. The rectosigmoid junction was included in the rectal subgroup. Patients with unknown tumour location (‘colon, not otherwise specified’ and ‘overlapping lesion of the colon’) were excluded from the tumour location analyses (*n* = 3283).

FCR obtains information on the cause of death from Statistics Finland, which in turn obtains the information from the deceased’s death certificate, which is filed by the treating physician. In addition, a specialist in forensic medicine approves all death certificates before they are ultimately registered.

### Statistical analysis

We analysed the median age at diagnosis using the interquartile range (IQR), gender distribution, tumour FCR classification and tumour location. Disease-specific survival (DSS) was calculated according to the Kaplan–Meier method using the log-rank analysis to determine the *p* value. We reported 95% confidence intervals (CIs) for 5-year survival and compared the CIs between groups. DSS was calculated as the time of diagnosis until death from CRC (registered cause of death C18–20, but excluding C18.1) or until the end of the follow-up period on 31 December 2016. Deaths unrelated to CRC (all other causes of deaths) were censored. Subgroup analyses were performed based on the tumour site and time periods. The relative survival analyses were performed using the relative survival estimation proposed by Ederer and Heise [12]. We compared the survival of these patients with survival among individuals matched for gender, age and time period from the population of Finland [13]. We considered *p* < 0.05 as statistically significant. All statistical analyses were performed using SPSS Statistics version 25 (IBM, Armonk, NY, USA).

### Permissions

The study protocol was approved by the National Institute of Health and Welfare (THL/722/5.05.00/2018).

## Results

This study included a total of 58 254 CRC patients (Supplementary Fig. 1). The median age at diagnosis was 71.9 years (IQR 62.9–79.7), with relatively equal gender and tumour location distributions (Supplementary Table 1). Median overall survival for all patients was 5.28 years, with data on all FCR classes appearing in Table 2. For FCR 1 (localised cancer), the median survival extended beyond our 11-year follow-up time period for this study.

**Table 2:** Median overall survival, 2006–2015 (95% CI^a^)

	**Overall survival (95% CI), in years**
**FCR classification**^**b**^	
FCR 0	4.06 (4.04–4.07)
FCR 1	NA^c^
FCR 2	6.50 (6.48–6.52)
FCR 3	2.15 (2.13–2.16)
FCR 4	3.44 (3.42–3.46)
FCR 5	8.85 (8.83–8.88)
FCR 6	1.07 (1.05–1.09)
All patients	5.29 (5.28–5.30)

*Abbreviations*: ^a^Confidence interval. ^b^Finnish Cancer Registry. 0) Unknown, 1) localised, 2) nonlocalised with only regional lymph node metastases, 3) metastasised further than to regional lymph nodes or invasion to adjacent tissues, 4) nonlocalised with no information on extent, 5) locally advanced with the tumour invading adjacent tissues and 6) nonlocalised with distant lymph node metastases as well. ^c^Not applicable, since the median survival for this specific category exceeded the duration of the follow-up time period for this study

### Five-year disease-specific survival

**Fig. 1:**
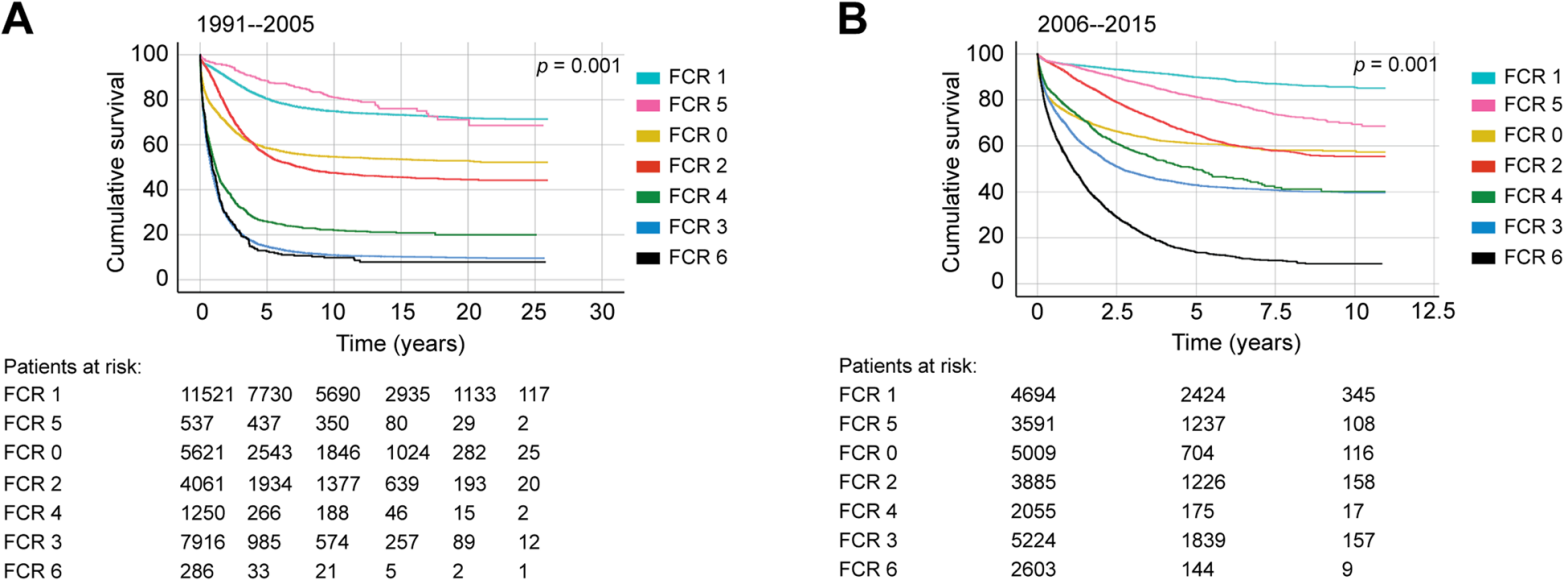
Disease-specific survival analysis of colorectal cancer patients diagnosed in **A**) 1991–2005 and **B**) 2006–2016. Finnish Cancer Registry classes: 0) unknown; 1) localised; 2) nonlocalised with only regional lymph node metastases; 3) metastasised further than to regional lymph nodes or invasion to adjacent tissues; 4) nonlocalised with no information on extent; 5) locally advanced with the tumour invading adjacent tissues; and 6) nonlocalised with distant lymph node metastases as well. *p* value for log-rank test

Overall, 5-year DSS for all CRC patients was 56.7% (95% CI 56.3–57.1%) for the period 1991 through 2015. The number of patients in each FCR class varied depending on how the categories were registered during different time periods. Since the early 1990s, both FCR classes 5 (locally advanced with the tumour invading adjacent tissues) and 6 (nonlocalised with distant lymph node metastases) have continued increasing, whereas FCR class 1 (localised) has continued declining (Supplementary Table 2A for colon cancer patients and Supplementary Table 2B for rectal cancer patients). We identified improvements in 5-year DSS for almost all FCR classes when comparing patients diagnosed between 1991 and 2005 with those diagnosed between 2006 and 2015 (Fig. 1).

For patients with localised disease (FCR 1), 5-year DSS was 80.5% (95% CI 80.1–81.7%) for the period 1991 through 2005 and 89.9% (95% CI 88.9–90.9%; Fig. 1) for the period 2006 through 2015. For patients with regional lymph node metastases (FCR 2), 5-year DSS improved from 55.5% (95% CI 53.9–57.1%) to 65.3% (95% CI 63.5–67.1%). During the period 2006 through 2015, 5-year DSS was 81.5% among patients with a locally advanced tumour invading an adjacent structure, but without lymph node or distant metastasis (FCR 5). Five-year DSS for patients with metastatic disease (FCR 6) was 12.6% (95% CI 8.7–16.5%) in the period 1991 through 2005, and 13.7% (95% CI 11.9–15.5%) for the period 2006 through 2015.

Five-year DSS appears to have steadily improved from the first time period (1991–1995) through to the last time period (2011–2015) for localised colon cancer, increasing from 79.2% to 91.4%, and for localised rectal cancer, increasing from 69.4% to 90.5% (FCR 1). This trend also emerged for patients with local lymph node metastasis (FCR 2) colon cancer, increasing from 49.1% to 62.0%, and rectal cancer, increasing from 44.0% to 68.9% (Tables 3 and 4). For patients with metastatic disease (FCR 6), 5-year DSS was 9.9% for colon cancer and 15.3% for rectal cancer for the period 2011 through 2015.

**Table 3:** Five-year disease-specific survival for colon cancer patients according to time period (95% CI^a^)

Time period	FCR^b^ class 0	FCR class 1	FCR class 2	FCR class 3	FCR class 4	FCR class 5	FCR class 6
1991–1995	50.7 (47.1–54.2)	79.2 (77.4–81.0)	49.1 (45.2–53.0)	10.7 (9.1–12.3)	19.4 (13.7–25.1)	NA^c^	NA
1996–2000	63.0 (60.2–65.7)	83.1 (81.5–84.7)	54.6 (51.1–58.1)	13.6 (11.8–15.4)	16.0 (10.7–21.3)	NA	NA
2001–2005	64.0 (60.9–67.1)	87.0 (85.6–88.4)	63.0 (59.9–66.1)	19.5 (17.5–21.5)	35.6 (30.7–40.5)	85.8 (81.2–89.9)	12.1 (7.4–16.8)
2006–2010	53.1 (49.4–56.8)	89.3 (87.7–90.9)	64.2 (61.1–67.3)	43.4 (41.4–45.4)	35.6 (29.7–41.5)	84.0 (81.5–86.5)	13.3 (10.4–16.2)
2011–2015	65.5 (63.0–68.0)	91.4 (89.2–93.6)	62.0 (58.5–65.5)	40.2 (36.3–44.1)	50.4 (45.3–55.5)	81.8 (78.7–84.9)	9.9 (6.6–13.2)

*Abbreviations*: ^a^Confidence interval. ^b^Finnish Cancer Registry. 0) Unknown, 1) localised, 2) nonlocalised with only regional lymph node metastases, 3) metastasised further than to regional lymph nodes or invasion to adjacent tissues, 4) nonlocalised with no information on extent, 5) locally advanced with the tumour invading adjacent tissues and 6) nonlocalised with distant lymph node metastases as well. ^c^Not applicable

**Table 4:** Five-year disease-specific survival for rectal cancer patients according to time period (95% CI^a^)

Time period	FCR^b^ class 0	FCR class 1	FCR class 2	FCR class 3	FCR class 4	FCR class 5	FCR class 6
1991–1995	53.2 (48.9–57.5)	69.4 (67.0–71.8)	44.0 (38.7–49.3)	12.2 (9.8–14.6)	14.9 (7.8–22.0)	NA^c^	NA
1996–2000	58.3 (55.0–61.6)	78.0 (75.8–80.2)	47.2 (42.9–51.5)	13.6 (11.2–16.0)	14.9 (7.5–22.3)	NA	NA
2001–2005	56.7 (53.0–60.4)	83.1 (81.1–85.1)	65.8 (61.9–69.7)	18.5 (16.0–21.0)	33.7 (27.4–40.0)	87.9 (82.8–93.0)	10.3 (4.0–16.6)
2006–2010	52.5 (48.1–56.8)	88.5 (86.7–90.3)	67.8 (64.1–71.5)	44.4 (41.7–47.1)	41.8 (32.3–50.8)	79.2 (75.7–82.7)	17.6 (13.1–22.1)
2011–2015	63.6 (60.5–66.7)	90.5 (87.8–93.2)	68.9 (64.6–73.2)	38.6 (33.3–43.9)	55.5 (48.6–62.4)	75.7 (70.8–80.6)	15.3 (11.0–19.6)

*Abbreviations*: ^a^Confidence interval. ^b^Finnish Cancer Registry. 0) Unknown, 1) localised, 2) nonlocalised with only regional lymph node metastases, 3) metastasised further than to regional lymph nodes or invasion to adjacent tissues, 4) nonlocalised with no information on extent, 5) locally advanced with the tumour invading adjacent tissues and 6) nonlocalised with distant lymph node metastases as well. ^c^Not applicable

### Five-year disease-specific survival according to tumour location and gender

Comparing the earlier time period with the later (1991–2005 vs. 2006–2015), 5-year DSS for right-sided colon cancer was 54.8% (95% CI 53.8–55.8%) versus 59.9% (95% CI 58.7–61.1%), 54.1% (95% CI 52.9–55.3%) versus 61.0% (95% CI 59.8–62.2%) for left-sided colon cancer and 53.6% (95% CI 52.2–55.0%) versus 62.3% (95% CI 61.3–63.3%) for rectal cancer.

**Fig. 2:**
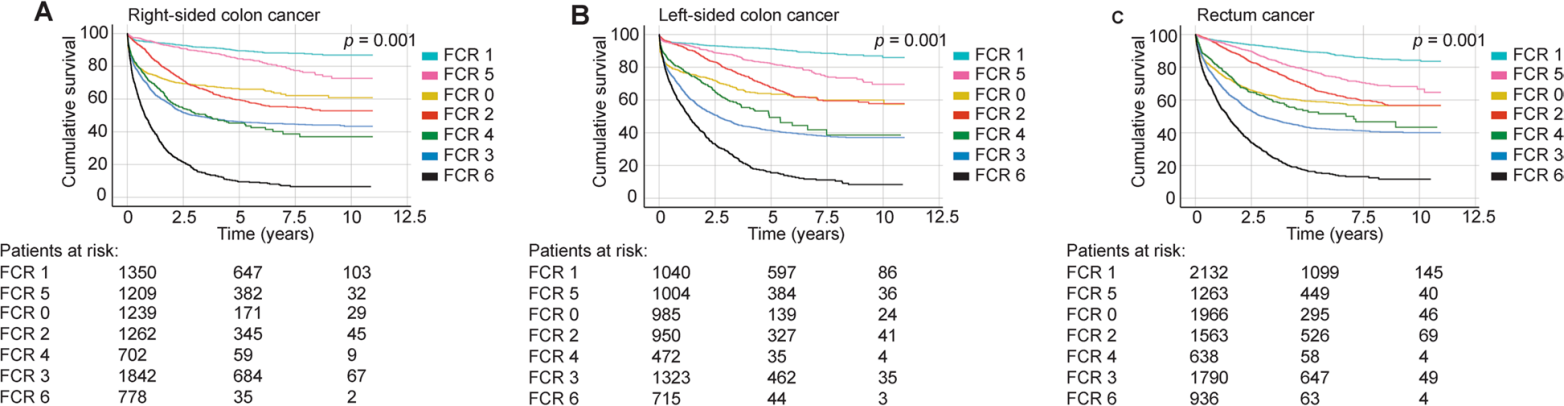
Disease-specific survival analysis according to the tumour locations. **A**) Right-sided colon cancer, **B**) left-sided colon cancer and **C**) rectal cancer among patients diagnosed in 2006–2015. Finnish Cancer Registry classes: 0) unknown; 1) localised; 2) nonlocalised with only regional lymph node metastases; 3) metastasised further than to regional lymph nodes or invasion to adjacent tissues; 4) nonlocalised with no information on extent; 5) locally advanced with the tumour invading adjacent tissues; and 6) nonlocalised with distant lymph node metastases as well. *p* value for log-rank test

In the period 2006 through 2015, 5-year DSS among patients diagnosed with localised disease (FCR 1) remained rather similar for those with right-sided colon cancer, left-sided colon cancer and rectal cancer (Fig. 2 and Table 5). Among patients with regional lymph node metastases (FCR 2), as well as among patients with metastatic cancer (FCR 6), 5-year DSS was worse among patients with right-sided disease (FCR 2: 59.6%; FCR 6: 9.5%) compared with those with left-sided colon cancer (FCR 2: 67.7%; FCR 6: 15.5%) or rectal cancer (FCR 2: 68.4%; FCR 6: 16.9%; Table 5).

**Table 5:** Five-year disease-specific survival according to tumour location, 2006–2015 (95% CI^a^)

	**All tumour sites**	**Right-sided colon**	**Left-sided colon**	**Rectum**
**FCR classification**^**b**^				
FCR 0	62.3 (60.5–64.1)	66.0 (62.9–69.1)	63.5 (59.8–67.2)	59.3 (56.8–61.8)
FCR 1	89.9 (88.9–90.9)	89.5 (87.7–91.3)	91.2 (89.4–93.0)	89.5 (88.1–90.9)
FCR 2	65.3 (63.5–67.1)	59.6 (56.5–62.7)	67.7 (64.2–71.2)	68.4 (65.7–71.1)
FCR 3	43.9 (42.5–45.3)	46.2 (43.8–48.6)	41.4 (38.7–44.1)	43.3 (40.9–45.7)
FCR 4	49.4 (46.3–52.5)	45.3 (40.6–50.0)	49.4 (42.5–56.3)	53.5 (48.4–58.6)
FCR 5	81.5 (79.9–83.1)	84.6 (82.1–87.1)	82.2 (79.5–84.9)	78.2 (75.6–80.9)
FCR 6	14.1 (12.3–15.9)	9.5 (7.0–12.0)	15.5 (12.2–18.8)	16.9 (14.0–19.8)
All patients	61.1 (60.5–61.7)	59.9 (58.7–61.1)	61.0 (59.6–62.4)	62.3 (61.3–63.3)

Abbreviations: ^a^Confidence interval. ^b^Finnish Cancer Registry. 0) Unknown, 1) localised, 2) nonlocalised with only regional lymph node metastases, 3) metastasised further than to regional lymph nodes or invasion to adjacent tissues, 4) nonlocalised with no information on extent, 5) locally advanced with the tumour invading adjacent tissues and 6) nonlocalised with distant lymph node metastases as well

When comparing the 5-year DSS for the time period 2006 through 2015 according to gender, there were no significant differences in survival (Supplementary Table 3).

### Relative survival

Comparing the time periods 1991–2005 with 2006–2015, relative survival improved over time (Table 6). For 2006 through 2015 patients with localised disease (FCR 1) exhibited a 5-year relative survival of 93.6%. For patients with regional lymph node metastases (FCR 2), 5-year relative survival was 68.2%. Among patients with a locally advanced tumour invading to an adjacent structure, but without lymph node or distant metastasis (FCR 5), 5-year relative survival was 84.2%. For patients with metastatic disease (FCR 6), 5-year relative survival was 14.0%. The 5-year relative survival among patients diagnosed with localised disease (FCR 1) with left-sided colon was better (97.4%; Supplementary Table 4), compared with patients with right-sided colon cancer (92.6%) and rectal cancer (92.1%). In addition, among patients with regional lymph node metastases (FCR 2) or metastatic disease (FCR 6), 5-year relative survival was worse among patients with right-sided disease compared with patients with left-sided colon cancer or rectal cancer (Supplementary Table 4).

**Table 6:** Five-year relative survival according to time period (95% CI^a^)

	**1991–2015**	**1991–2005**	**2006–2015**
**FCR classification**^**b**^			
FCR 0	62.4 (60.9–63.9)	62.6 (60.7–64.5)	63.3 (60.8–65.6)
FCR 1	87.2 (86.1–88.2)	84.9 (83.7–86.1)	93.6 (91.5–95.3)
FCR 2	62.7 (61.2–64.2)	58.1 (56.1–60.1)	68.2 (65.9–70.4)
FCR 3	27.8 (26.9–28.7)	15.4 (14.5–16.4)	46.3 (44.6–48.1)
FCR 4	41.0 (38.6–43.4)	25.7 (22.8–28.6)	51.9 (48.2–55.5)
FCR 5	86.2 (83.8–88.3)	96.2 (87.6–98.9)	84.2 (81.5–86.5)
FCR 6	13.9 (12.3–15.6)	12.7 (8.9–17.3)	14.0 (12.2–15.8)
All patients	59.9 (59.3–60.4)	57.0 (56.3–57.8)	63.4 (62.6–64.3)

*Abbreviations*: ^a^Confidence interval. ^b^Finnish Cancer Registry. 0) Unknown, 1) localised, 2) nonlocalised with only regional lymph node metastases, 3) metastasised further than to regional lymph nodes or invasion to adjacent tissues, 4) nonlocalised with no information on extent, 5) locally advanced with the tumour invading adjacent tissues and 6) nonlocalised with distant lymph node metastases as well

## Discussion

In this study, we aimed to determine disease-specific (DSS) and relative survival among 58 254 patients with CRC in Finland. We found that, over time, when comparing patients diagnosed between 1991 and 2005 with those diagnosed between 2006 and 2015, both DSS and relative survival improved. Cancer-specific data on staging received from FCR relies on FCR’s own classification for tumour spread [10]. It is, however, fairly comparable to the TNM staging classification. As such, we aimed to explore survival according to the FCR staging classification. We noted a clear improvement among most FCR classes.

Our results for 5-year relative survival among patients diagnosed in the period 2006 through 2015 with local, regional and metastatic CRC (FCR 1, 2 and 6) agree with population-based studies from the US, the Netherlands and Australia [14–16]. The seemingly better survival reported in the Dutch study may result in part from patient selection, since the study included only patients with an endoscopic or operative treatment intent. In general, 5-year DSS and relative survival among Finnish CRC patients diagnosed between 2006 and 2015 appear consistent with results from other Western countries.

For patients with locally advanced disease with the tumour invading adjacent tissues (FCR 5), both 5-year DSS and relative survival were seemingly better in the earlier time period. However, this may be explained by the low volume of patients in the earlier time periods (Tables 3–4; Supplementary Tables 2A–B). Because these locally advanced cases, which were previously more likely registered as metastasised further than the regional lymph nodes or invasion to adjacent tissues (FCR 3), were increasingly registered as FCR 5, we can speculate that 5-year DSS ultimately reached its actual level. During the period 2006 through 2015, 5-year DSS for FCR 5 – that is, the group comparable to TNM stage II – agrees with findings from an Australian study [15]. However, a Dutch study observed a clearly better 5-year relative survival among endoscopically or operatively treated patients [16].

FCR classes 3 (metastasised further than the regional lymph nodes or invasion to adjacent tissue) and 4 (nonlocalised with no information on extent) represent problematic groups, roughly corresponding to TNM stages II through IV and III through IV, respectively, as shown in Table 1. TNM stages II and III generally exhibit better survival and, therefore, FCR classes 3 and 4 have clearly better survival than FCR 6, which corresponds only to TNM stage IV. Therefore, FCR classes 3 and 4 are difficult to extrapolate to clinical settings and, also difficult to reliably compare to TNM staging.

For patients with localised disease (FCR 1), survival seems similar when comparing right-sided colon, left-sided colon and rectal cancers. For lymph node-positive (FCR 2) and metastatic cancer (FCR 6) patients, we found that 5-year DSS was worse among those with right-sided colon cancer compared with patients with left-sided colon or rectal cancer. Similar findings were noted in a systematic review [17] and in another study, where researchers found that patients with stage I and III (FCR 1 and 2) right-sided colon cancer exhibited a survival consistent with our results [18]. The reasons may stem from surgical technique differences, differences in embryonic evolution or differences in the microbiome between the right and left side of the colon [11, 19, 20]. Thus, colon cancer patients with right-sided disease in general exhibited a slightly worse prognosis compared with patients with left-sided disease even at the same stage.

In general, improved survival may result from stage migration when patients over time are staged more accurately and, hence, survival across all of the staging classes impacted improves. The reliable staging of CRC requires at least 12 regional lymph nodes for pathological examination. Pathological reporting based on less than 12 regional lymph nodes results in low-quality staging and represents a poor prognostic marker itself [21–23]. Modern pathological reporting relying on 12 lymph nodes gradually emerged as a method around 2008 in Finland, providing more accurate staging determination and partly explaining the improved survival we observed here. In the past, surgery was less extensively performed when stage III disease was in some cases considered local (for example stage II). This also might explain why localised disease survival has improved in recent years. Moreover, a systematic reporting form has been introduced into pathology departments as good medical practice [24]. In addition, modern adjuvant and neoadjuvant treatment have also improved in recent years, particularly as the treatment of patients with metastatic disease has become more individualised. This also explains the improved results especially related to survival for rectal cancer patients. Furthermore, enhanced recovery after surgery (ERAS) protocols have been implemented, and multidisciplinary teams have been involved in the care of patients. For instance, one Swedish study on colon cancer showed that the overall 5-year survival improved among those undergoing surgery between 2007 and 2010 for procedures performed by subspecialist colorectal surgeons when compared with surgery performed by general surgeons (60% vs. 48%) [25]. In Finland, however, surgery for colon and rectal cancers has only recently and gradually been centralised, a shift not yet reflected in the results among patients diagnosed between 2006 and 2015.

One strength of our study lies in the large cohort of data from FCR, which records information on every cancer case in Finland and features an excellent coverage. However, we must regard the FCR classification of tumour spread as a limitation, given that it is difficult to compare these data precisely with data relying on the UICC TNM staging. Despite this limitation, the use of this kind of classification also allows for the inclusion of cases with incomplete information. Recently, one study showed that the FCR classification agrees fairly well with the TNM staging classification [10].

## Conclusions

In conclusion, this population-based study in Finland investigated current survival among CRC patients according to tumour spread, comparable to the TNM staging classification. We found that survival has improved in nearly all subgroups in recent decades, mirroring findings from other Western countries. We confirmed that survival among colon cancer patients with right-sided disease is generally worse when compared with left-sided disease. In future, these results may be used as a reference when evaluating local treatment outcomes. However, caution must be taken when comparing the FCR classification with the TNM staging. Recording stage-specific information for patients’ cancers remains of utmost importance. Doing so aids both clinical and international comparisons, particularly if data from FCR can be retrieved according to the UICC TNM staging classification.

## Supplementary Information

**Additional file 1.**

**Additional file 2.**

**Additional file 3.**

**Additional file 4.**

**Additional file 5.**

## Data Availability

Due to the large series of datasets from FCR, we are not permitted to release the data in its current form.

## Abbreviations

CRC: Colorectal cancer
DSS: Disease-specific survival
CI: Confidence interval
FCR: Finnish Cancer Registry
IQR: Interquartile range
UICC TNM: Union for International Cancer Control Tumour Node Metastasis
