# Supplementary figures and images for: Long-term survival among colorectal cancer patients in Finland, 1991–2015: a nationwide population-based registry study
Source: BMC Cancer. 2022 Apr 2;22:356. doi: 10.1186/s12885-022-09460-0 (PMC8976396; doi:10.1186/s12885-022-09460-0)

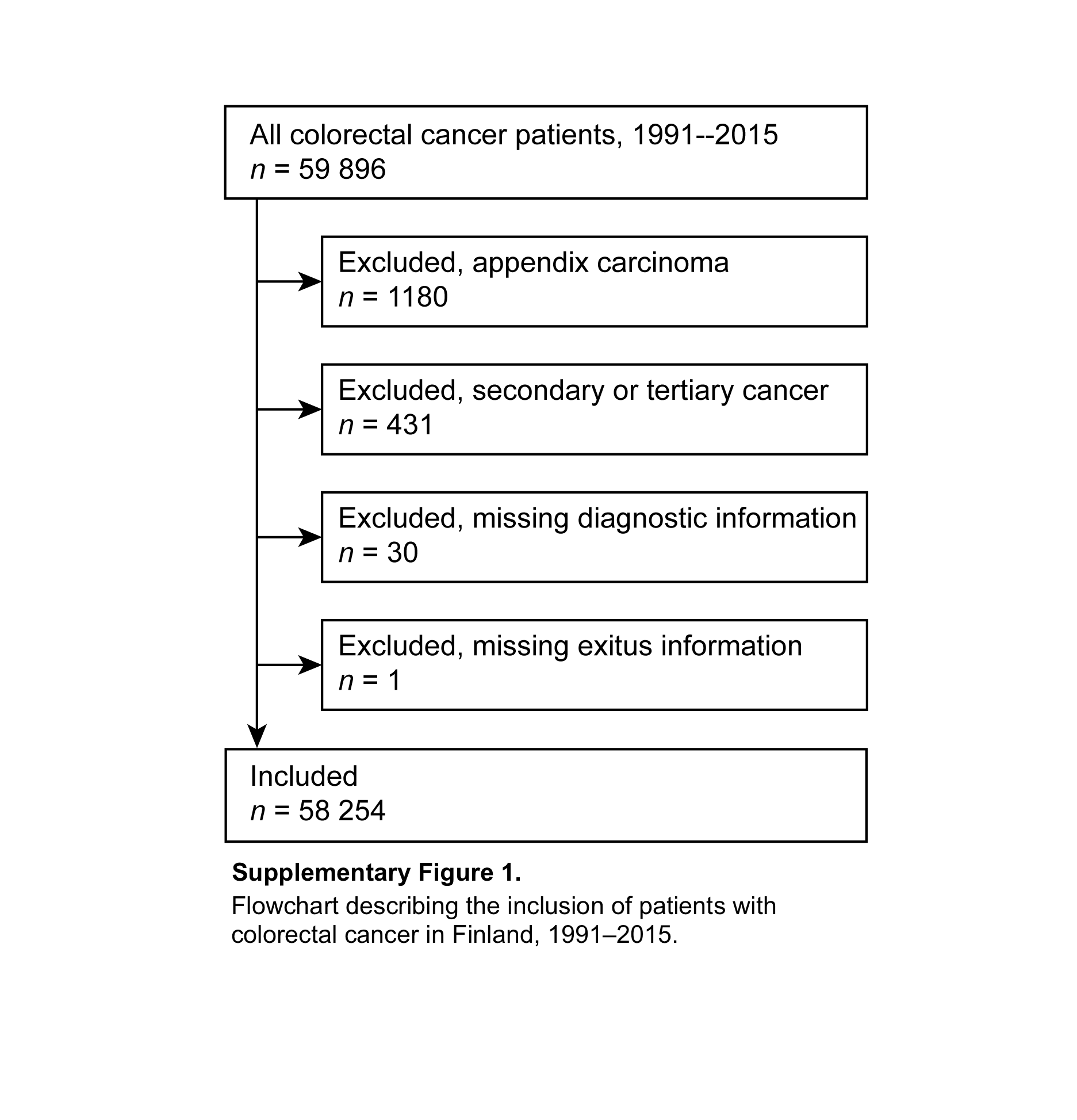

Supplement: Supplementary file 1 — Additional file 1. [file 12885_2022_9460_MOESM1_ESM.tiff]
